# Alginate Oligosaccharides Ameliorate DSS-Induced Colitis through Modulation of AMPK/NF-κB Pathway and Intestinal Microbiota

**DOI:** 10.3390/nu14142864

**Published:** 2022-07-13

**Authors:** Yue Zhang, Congcong Guo, Yanru Li, Xianlei Han, Xuegang Luo, Liehuan Chen, Tongcun Zhang, Nan Wang, Weiming Wang

**Affiliations:** 1 School of Traditional Chinese Medicine, Southern Medical University, Guangzhou 510515, China; 2 Key Laboratory of Industrial Fermentation Microbiology, Ministry of Education and Tianjin, College of Biotechnology, Tianjin University of Science and Technology, Tianjin 300457, China; geby555@163.com (Y.Z.); gcongu5413@163.com (C.G.); yanrulee930620@mail.tust.edu.cn (Y.L.); Hxl17302297526@163.com (X.H.); luoxuegang@hotmail.com (X.L.); tony@tust.edu.cn (T.Z.); 3 Tianjin Engineering Research Center of Microbial Metabolism and Fermentation Process Control, Tianjin 300457, China; 4 College of Animal Sciences and Technology, Zhongkai Agricultural Engineering College, Guangzhou 510225, China; chitins@126.com; 5 Guangzhou Youlan Marine Biological Technology Co., Ltd., Guangzhou 510530, China

**Keywords:** alginate oligosaccharides, AMPK, intestinal microbiota, NF-κB, ulcerative colitis

## Abstract

Alginate oligosaccharides (AOS) are shown to have various biological activities of great value to medicine, food, and agriculture. However, little information is available about their beneficial effects and mechanisms on ulcerative colitis. In this study, AOS with a polymerization degree between 2 and 4 were found to possess anti-inflammatory effects in vitro and in vivo. AOS could decrease the levels of nitric oxide (NO), IL-1β, IL-6, and TNFα, and upregulate the levels of IL-10 in both RAW 264.7 and bone-marrow-derived macrophage (BMDM) cells under lipopolysaccharide (LPS) stimulation. Additionally, oral AOS administration could significantly prevent bodyweight loss, colonic shortening, and rectal bleeding in dextran sodium sulfate (DSS)-induced colitis mice. AOS pretreatment could also reduce disease activity index scores and histopathologic scores and downregulate proinflammatory cytokine levels. Importantly, AOS administration could reverse DSS-induced AMPK deactivation and NF-κB activation in colonic tissues, as evidenced by enhanced AMPK phosphorylation and p65 phosphorylation inhibition. AOS could also upregulate AMPK phosphorylation and inhibit NF-κB activation in vitro. Moreover, 16S rRNA gene sequencing of gut microbiota indicated that supplemental doses of AOS could affect overall gut microbiota structure to a varying extent and specifically change the abundance of some bacteria. Medium-dose AOS could be superior to low- or high-dose AOS in maintaining remission in DSS-induced colitis mice. In conclusion, AOS can play a protective role in colitis through modulation of gut microbiota and the AMPK/NF-kB pathway.

## 1. Introduction

As a chronic inflammation disease in the colon, ulcerative colitis (UC) is characterized by fever, vomiting, abdominal pain, diarrhea, bloody mucopurulent stools, and digestive disorders. Due to the pro-neoplastic effect of chronic intestinal inflammation, colitis patients, if not treated in time, have a significantly increased risk of colorectal cancer. Currently, the cause of UC is still unclear, but genetic, dietary, and environmental factors may contribute to UC risk. Clinically, UC is mainly treated with conventional therapy of corticosteroids and immunomodulators, as well as biological therapy such as anti-tumor necrosis factor (TNF) agents [1]. However, such long-term treatment may cause multiple side effects and a high recurrence rate, coupled with high costs. Therefore, researchers have paid increasing attention to alternative treatments with fewer adverse effects, such as functional foods and dietary supplements derived from natural resources.

Alginate, an acidic polysaccharide extracted from marine brown algae, is a whole family of linear copolymers containing blocks of (1,4)-linked β-D-mannuronate (M) and α-l-guluronate (G) residues [2]. G and M are linked through homologous forms, such as consecutive G or M residues (GGGGGG or MMMMMM), or through heterologous forms, such as alternating M and G residues (GMGMGM). Additionally, alginate can be degraded to alginate oligosaccharides (AOS) with a lower molecular weight, lower viscosity, and better solubility and bioavailability [3]. AOS have been reported to possess multiple biological activities, such as antioxidant [4], antibacterial [5] anti-inflammatory [6], antiapoptotic [7], antiobesity [8,9], and antitumor activities [10]. Due to their antioxidant activities, AOS have been considered to have protective effects on the myocardial injury induced by doxorubicin [11] or ischemia/reperfusion [12]. The beneficial effects of AOS on the host also involve reducing the risks of cardiovascular disease [13,14] and inhibiting neuroinflammation [15]. Recently, AOS were reported to prevent small-intestine mucositis induced by busulfan [16], as well as ameliorate intestinal damage and inflammation in fumonisin B1 (FB1)-treated mice [17] and enterotoxigenic *Escherichia coli* (ETEC)-infected weaned pigs [18]. However, the beneficial effect and mechanism of AOS on ulcerative colitis have not been fully explored.

Adenosine 5′-monophosphate-activated protein kinase (AMPK) is a key sensor of cellular energy status [19]. Recent studies indicate that AMPK signaling performs a vital role in inhibiting inflammatory responses. AMPK β1 subunit genetic deletion in bone-marrow-derived macrophages (BMDMs) isolated from global AMPKβ1^−/−^ mice was shown to decrease AMPK α1 activity and increase the levels of proinflammatory cytokines, such as tumor necrosis factor-α (TNF-α), interleukin-1β (IL-1β), interleukin-6 (IL-6), etc. [20]. Using macrophage-specific AMPKβ1-deficient mice, Banskota et al. demonstrated that AMPKβ1 deletion could promote nuclear factor-κB (NF-κB) nuclear translocation, thus upregulating the production of proinflammatory cytokines and exacerbating the severity of dextran sodium sulfate (DSS)-induced colitis in mice [21]. Meanwhile, AMPK activation (phosphorylation on Thr-172) was shown to exert a potential therapeutic effect in animal models with colitis [22], pneumonia [23], and neuritis [24]. Additionally, accumulating evidence also suggested the anti-inflammatory function of activated AMPK in lipopolysaccharide (LPS)-stimulated murine macrophage RAW 264.7 cells [25,26,27]. Studies have also indicated that AMPK activation could inhibit LPS-induced NF-κB signaling pathway activation, thereby reducing NF-κB-dependent proinflammatory cytokine production in RAW 264.7 cells [6,25,26]. However, whether AOS can activate AMPK in UC is still unknown.

Microbial dysbiosis is closely related to colitis in humans and experimental animals [28]. Gut microbiota imbalance plays an essential role in colitis pathogenesis by affecting the metabolic pathway, gut barrier, and immunity [29]. Studies have shown the abundant decrease in *Bacteroidetes* and the abundant increase in *Proteobacteria* and *Actinobacteria* in the fecal microbiota of UC patients [30,31]. In the cecal contents of DSS-induced colitis mice, *Proteobacteria* and *Bacteroidetes* showed a significant increase and decrease in their relative abundance, respectively [32,33]. Recently, unsaturated alginate oligosaccharides (UAOS) have been reported to attenuate high-fat-diet (HFD)-induced obesity through gut microbiota modulation [9]. However, how AOS affect gut microbiota in UC remains elusive.

This study aimed to investigate the anti-inflammatory function and mechanism of AOS on UC in DSS-induced colitis mice in vivo and LPS-stimulated RAW 264.7 cells and BMDM in vitro. AOS treatment was found to inhibit LPS-stimulated inflammatory responses in vitro via AMPK activation and NF-κB repression. Meanwhile, AOS administration could alleviate colitis in vivo through modulation of intestinal microbiota and the AMPK/NF-κB pathway.

## 2. Materials and Methods

### 2.1. Cell Culture and Treatment

From the American Type Culture Collection (Rockville, MD, USA), murine RAW 264.7 cells were obtained and cultured at 37 °C with a humidified atmosphere of 5% CO_2_/95% air in Dulbecco’s modified Eagle’s medium (DMEM) (12100061, GIBCO BRL, Grand Island, NY, USA.) containing 10% fetal bovine serum (FBS) (AusGeneX, Brisbane, Australia), 100 U/mL penicillin, and 100 μg/mL streptomycin. From the tibia and femur of C57BL/6J mice, BMDMs were isolated and cultured in DMEM medium containing 10% FBS and 25 ng/mL macrophage colony-stimulating factor (M-CSF), as previously described [34]. From Guangzhou Youlan Marine Biotechnology Co., Ltd. (Guangzhou, China), AOS (DP: 2-4) were obtained and prepared as previously reported [35]. LPS (*E. coli*, O55: B5) was obtained from Sigma-Aldrich (St. Louis, MO, USA). AOS were dissolved in the culture media at various concentrations (125, 250 or 500 μg/mL), followed by preincubating cells separately in the culture media and then stimulating the cells with LPS (1 μg/mL) for the indicated time points.

### 2.2. Cell Viability Detection

From Solarbio Science and Technology Co., Ltd. (Beijing, China), MTT reagent (3-(4,5-dimethylthiazol-2-yl)-2,5-diphenyltetrazolium bromide) was obtained and used to assay the viability of RAW 264.7 and BMDM cells. Briefly, after seeding into 96-well plates at 2 × 10^5^ cells/well, RAW 264.7 and BMDM cells were preincubated with AOS at 0, 125, 250, or 500 μg/mL for 24 h, followed by stimulation with or without 1 µg/mL LPS for another 24 h, and replenishing each well with 10 µL MTT solution (5 mg/mL in PBS). After incubation for another 4 h, the supernatant was removed, and each well was supplemented with 100 µL DMSO for dissolution of formazan crystals. Finally, the absorbance was measured at 490 nm using a microplate reader (BioTek, Winooski, VT, USA).

### 2.3. Measurement of Nitric Oxide (NO) and Cytokines

For measuring NO, RAW 264.7 cells were seeded at 2 × 10^5^ cells per well in 96-well plates, followed by preincubation for 24 h with AOS at 0, 125, 250, or 500 μg/mL and then stimulation with 1 µg/mL LPS for another 24 h. NO production in cellular supernatants was analyzed using the NO assay kit (Beyotime, Shanghai, China). The mixture absorbance was measured spectrophotometrically at 540 nm using a microplate reader (BioTek, Winooski, VT, USA).

For measuring cytokines, RAW 264.7 and BMDM cells were preincubated for 24 h with AOS at 0, 125, 250, or 500 μg/mL and then stimulated with 1 µg/mL LPS for another 24 h. IL-1β, IL-6, TNF-α, and IL-10 levels were evaluated using an enzyme-linked immunosorbent assay (ELISA) kit (R&D Systems, Minneapolis, MN, USA) as instructed by the manufacturer.

### 2.4. Animal Assay

From the Experimental Animal Center of Military Medical College (Beijing, China), 8-week-old male C57BL/6 mice were obtained and housed in a 12 h light/dark cycle with ad libitum food and water. All animal experiments were approved by the Institutional Animal Care and Use Committee, Tianjin University of Science and Technology (No. 20201107CB) and performed based on the National Institutes of Health Guide for the Care and Use of Laboratory Animals (the 8th Edition, revised in 2011). The mice were allotted to five groups (n = 6 per group): Control group (standard diet), DSS group (DSS challenge), AOS-L group (DSS challenge + 400 mg/kg AOS), AOS-M group (DSS challenge + 600 mg/kg AOS), and AOS-H group (DSS challenge + 800 mg/kg AOS). DSS-colitis grade (36,000–50,000 MW) was obtained from MP Biomedicals (Solon, OH, USA). Mice were orally treated with AOS (AOS-L, AOS-M, and AOS-H groups) or PBS (Control and DSS groups) once daily for 14 days. Colitis induction was performed by adding DSS (2.5% *w*/*v*) to sterile drinking water in the experiment from Days 8 to 14. During the experiment, the body weight of each animal was measured every day. On Day 15, mice were sacrificed to yield samples for subsequent experiments. The disease activity index (DAI) scores of mice were evaluated by monitoring pathological features, including stool consistency, bloody stool, weight loss, etc. Blood samples were collected using the retro-orbital bleeding method, and serum samples were obtained by centrifugation at 4 ℃ and 1500 rpm for 30 min. The levels of IL-1β, IL-6, TNF-α, and IL-10 in the serum samples were analyzed using ELISA kits (R&D Systems, Minneapolis, MN, USA), and the IFN-γ levels in the serum samples were also analyzed using an ELISA kit (ZCIBIO Technology Co., Ltd., Shanghai, China).

### 2.5. Hematoxylin and Eosin (H&E) Staining

Briefly, fresh ileal and colonic tissues were cut into sections and fixed in 4% paraformaldehyde, followed by dehydration in a graded ethanol series, embedding in paraffin, cutting into 5 μm slices, and H&E staining for pathological assessment. The histopathological analysis was performed according to the criteria of Chiu′s score [36].

### 2.6. Western Blotting Detection

For measuring the expressions of p-P65/P65 and p-AMPK/AMPK, RAW 264.7 and BMDM cells were preincubated for 24 h with AOS at 0, 125, 250, or 500 μg/mL and then stimulated with 1 µg/mL LPS for another 1 h. The preparation of total protein from cells or colonic tissues was performed by using RIPA lysis buffer (MedChemExpress LLC, Princeton, NJ, USA). After separation through 10% SDS-PAGE, the samples were transferred to a nitrocellulose membrane, followed by blocking for 1 h at room temperature with 5% nonfat milk in Tris-buffered saline containing 0.1% Tween 20 (TBST) and then incubation overnight at 4 °C with the following primary antibodies: anti-p65 (sc-514451, Santa Cruz Biotechnology, Inc., Santa Cruz, CA, USA) (dilution: 1:500), anti-phospho-p65 (sc-136548, Santa Cruz Biotechnology, Inc., Santa Cruz, CA, USA) (dilution: 1:500), anti-p-AMPK (2535S, Cell Signaling Technology, Inc., Boston, MA, USA) (dilution: 1:1000), anti-AMPK (sc-74461, Santa Cruz Biotechnology, Inc., Santa Cruz, CA, USA) (dilution: 1:500), and anti-β-actin (UM4001, UtiBody, Tianjin, China) (dilution: 1:5000). After 3 washes with TBST, the membranes were incubated at room temperature for 2 h with the secondary antibodies of IRDye-680RD IgG (926-68071, Li-COR Biosciences, Lincoln, NE, USA) (dilution: 1:5000), or IRDye-800CW IgG (926-32210, Li-COR Biosciences, Lincoln, NE, USA) (dilution: 1:5000). Finally, protein bands were visualized using an Odyssey System (Li-COR Biosciences, Lincoln, NE, USA), with β-actin as an internal control. Data were quantified with ImageJ software (U.S. National Institutes of Health, Bethesda, MD, USA).

### 2.7. Intestinal Microbiota Analysis by High-Throughput Sequencing

Intestinal microbiota were analyzed by OE Biotech Co., Ltd. (Shanghai, China). Extraction of total microbial genomic DNA from colonic and cecal contents was performed using the DNeasy PowerSoil Kit as directed by the manufacturer. Amplification of the V3-V4 hypervariable regions of the bacterial 16S rDNA gene was performed using 343F-5’-TACGGRAGGCAGCAG-3’; 798R-5’-AGGGTATCTAATCCT-3’ in a thermocycler PCR system (Bio-Rad 580BR10905, Hercules, CA, USA), followed by sequencing using Illumina Novo seq6000. Finally, Vsearch software was used for primer sequence removal and assembly of clean reads to generate operational taxonomic units (OTUs) with a 97% similarity cutoff.

### 2.8. Statistical Analysis

Multiple comparisons between groups were performed by one-way ANOVA, followed by Tukey’s test in GraphPad Prism 6.0 (Graph Pad Software Inc., San Diego, CA, USA). Data were shown as mean ± standard deviation (SD), with significance shown as ^#^ *p* < 0.05, ^##^ *p* < 0.01, ^###^ *p* < 0.001, ^####^ *p* < 0.0001 relative to the Control group and * *p* < 0.05, ** *p* < 0.01, *** *p* < 0.001, **** *p* < 0.0001 relative to the LPS or DSS group.

## 3. Results

### 3.1. AOS Anti-Inflammatory Activity

**Figure 1:**
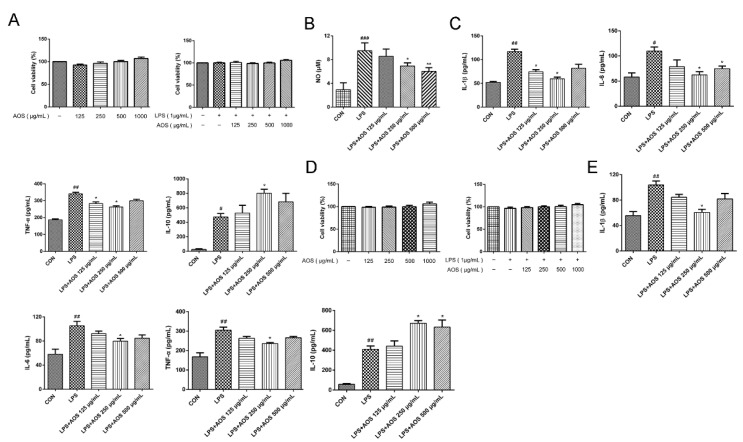
AOS anti-inflammatory activity in LPS-induced RAW 264.7 and BMDM cells. (**A**) MTT assay of viability in RAW 264.7 cells. RAW 264.7 cells were preincubated separately with AOS at 125, 250, and 500 μg/mL for 24 h, followed by stimulation with or without 1 μg/mL LPS for another 24 h. (**B**) NO production in RAW 264.7 cells as tested by NO assay kit. (**C**) ELISA analysis of IL-1β, IL-6, TNF-α, and IL-10 levels in RAW 264.7 cells. (**D**) MTT assay of viability in BMDMs. BMDMs were preincubated separately with AOS at 125, 250, and 500 μg/mL for 24 h, followed by stimulation with or without 1 μg/mL LPS for another 24 h. (**E**) ELISA analysis of IL-1β, IL-6, TNF-α and IL-10 levels in BMDMs. ^#^ *p* < 0.05, ^##^ *p* < 0.01, ^###^ *p* < 0.001 vs. Control group, * *p* < 0.05, ** *p* < 0.01 vs. LPS group, n = 3.

AOS anti-inflammatory activity was investigated by stimulating RAW 264.7 and BMDM cells with LPS as in vitro inflammatory models. The cytotoxic effect of AOS on RAW 264.7 cells was first investigated by preincubating RAW 264.7 cells with AOS at 125, 250, and 500 μg/mL for 24 h, followed by stimulation with or without 1 μg/mL LPS for 24 h. In Figure 1A, cell proliferation was seen to be unaffected by 125–500 μg/mL AOS. Meanwhile, the effect of AOS on the LPS-stimulated production of NO, proinflammatory cytokines (IL-1β, IL-6, and TNF-α), and anti-inflammatory cytokine (IL-10) was also examined. In Figure 1B,C, compared with the Control group, LPS stimulation was seen to increase the levels of NO, IL-1β, IL-6, TNF-α, and IL-10 significantly (*p* < 0.05, *p* < 0.01, and *p* < 0.001). Compared with LPS treatment alone, AOS treatment (250 and 500 μg/mL) could significantly inhibit NO production and IL-6 secretion (*p* < 0.05 and *p* < 0.01), and AOS treatment (125 and 250 μg/mL) could remarkably decrease the levels of IL-1β and TNF-α (*p* < 0.05). Additionally, 250 μg/mL AOS could further enhance IL-10 secretion relative to LPS treatment alone (*p* < 0.05). In Figure 1D, 125–500 μg/mL AOS was seen to have no effect on BMDM cell proliferation. In Figure 1E, AOS treatment was observed to obviously reduce the proinflammatory cytokine release and induce IL-10 production in LPS-stimulated BMDMs relative to LPS treatment alone (*p* < 0.05). These results indicated that AOS had potent anti-inflammatory activity in vitro.

### 3.2. Oral AOS Administration Attenuated Colitis in DSS-Treated Mice

**Figure 2:**
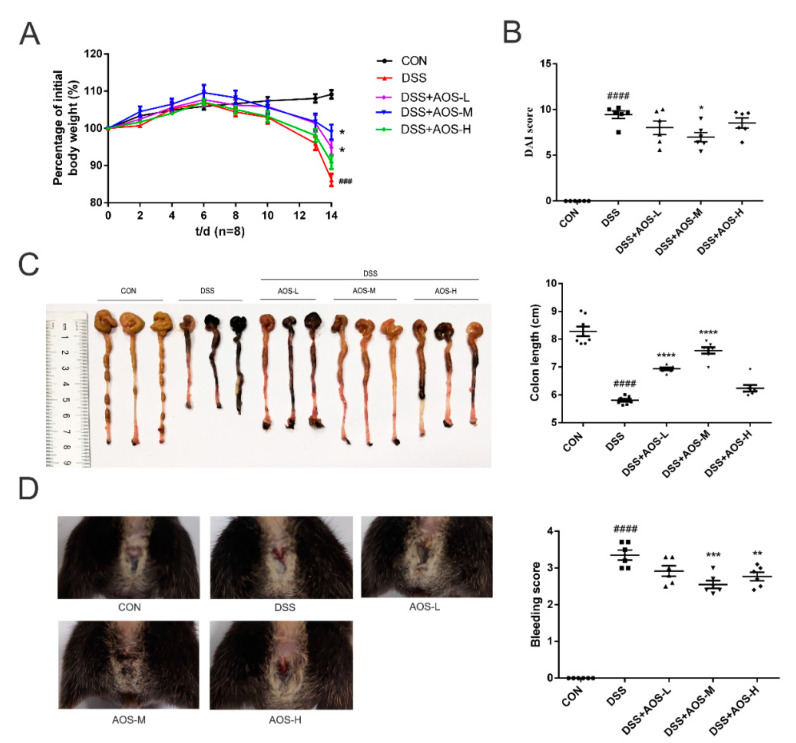
Effects of oral AOS administration on DSS-induced colitis in mice. The mice were gavaged with either PBS or AOS daily for 14 d, and colitis was induced from Days 8 to 14 by adding DSS (2.5% *w*/*v*) to sterile drinking water. (**A**) Changes in body weight. (**B**) Changes in DAI score. (**C**) Changes in colon length. (**D**) Changes in rectal bleeding. * *p* < 0.05, ** *p* < 0.01, *** *p* < 0.001, **** *p* < 0.0001 vs. DSS group; ^###^ *p* < 0.001, ^####^ *p* < 0.0001 vs. Control; (n = 6).

Next, a DSS-induced colitis mice model was established to investigate the anti-inflammatory effect of AOS in vivo. Whether AOS could relieve the symptoms of acute colitis in mice was investigated in terms of weight loss, colon shortening, rectal bleeding, and DAI score. In Figure 2A, DSS-challenged mice were seen to have a significant decrease in body weight, while the administration of AOS-L and AOS-M could obviously reduce body weight induced by DSS (^###^ *p* < 0.001 vs. Control group; * *p* < 0.05 vs. DSS group). Meanwhile, the DAI score was significantly lower in the AOS-M group than in the DSS group or the Control group (* *p* < 0.05 vs. DSS group; ^####^ *p* < 0.0001 vs. Control group) (Figure 2B). In Figure 2C, DSS-induced colon shortening was also seen to be significantly improved through oral administration of AOS-L and AOS-M (**** *p* < 0.0001 vs. DSS group; ^####^ *p* < 0.0001 vs. Control group). Oral administration of AOS-M and AOS-H was also shown to significantly improve rectal bleeding (Figure 2D) (** *p* < 0.01, *** *p* < 0.001 vs. DSS group; ^####^ *p* < 0.0001 vs. Control group). These results indicated that AOS could exert prominent intestinal protection in DSS-induced colitis mice.

### 3.3. Oral AOS Administration Ameliorated Histological Damages in Colonic and Ileal Tissues

**Figure 3:**
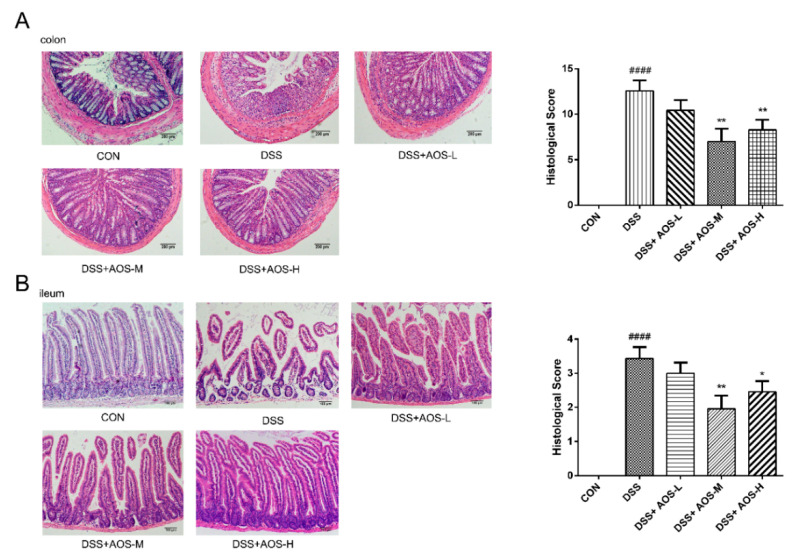
Effects of AOS on histological changes in colonic and ileal tissues. (**A**) Histopathological images and scores of colonic tissues; scale bar = 200 μm. (**B**) Histopathological images and scores of ileal tissues; scale bar = 100 μm. * *p* < 0.05, ** *p* < 0.01 vs. DSS group; ^####^ *p* < 0.0001 vs. Control group; n = 3.

The histological damages to the colon and ileum were investigated by H&E staining. In Figure 3A, DSS treatment was seen to induce marked loss of colonic mucosa, disruption or disappearance of glands, and infiltration of lamina propria inflammatory cells. However, these histological damages were improved in all the three AOS groups, especially the AOS-M and AOS-H groups (** *p* < 0.01 vs. DSS group). In Figure 3B, DSS treatment was also observed to induce ileal damage and villus height reduction, while these abnormal changes could be obviously alleviated by AOS administration (* *p* < 0.05, ** *p* < 0.01 vs. DSS group).

### 3.4. AOS Protected Mice against DSS-Induced Host Inflammation

**Figure 4:**
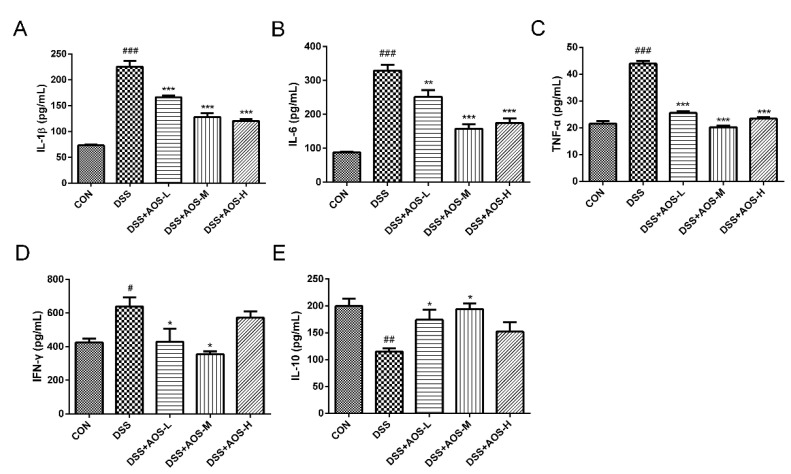
Effects of AOS on the production of proinflammatory cytokines in vivo as detected by ELISA analysis of the levels of IL-1β (**A**), IL-6 (**B**), TNF-α (**C**), IFN*-*γ (**D**), and IL-10 (**E**) in the serum of DSS-induced acute colitis mice. * *p* < 0.05, ** *p* < 0.01, *** *p* < 0.001 vs. DSS group; ^#^ *p* < 0.05, ^##^ *p* < 0.01, ^###^ *p* < 0.001 vs. Control group; n = 6.

The effects of AOS on proinflammatory cytokines were further investigated by ELISA analysis of IL-1β, IL-6, TNF-α, IFN*-*γ, and IL-10 levels in the serum of DSS-induced colitis mice. In Figure 4A–D, DSS challenge was seen to significantly increase the levels of TNF-α, IL-1β, IL-6, and IFN*-*γ (^#^ *p* < 0.05, ^###^ *p* < 0.001 vs. Control group). Meanwhile, DSS treatment was shown to significantly inhibit the IL-10 level (^##^ *p* < 0.01 vs. Control group) (Figure 4E). However, these changes were restored by AOS treatment. Compared with the DSS group, the three AOS groups could significantly reduce the secretion of IL-1β, IL-6, and TNF-α (*p* < 0.01 and *p* < 0.001). AOS-M and AOS-H treatment could downregulate the IFN-γ level and upregulate the anti-inflammatory cytokine IL-10 level in the serum of DSS-induced colitis mice (*p* < 0.05), suggesting that AOS also have anti-inflammation ability in vivo.

### 3.5. AOS Suppressed DSS-Induced Inflammation In Vivo through AMPK/NF-κB Signaling Pathway

**Figure 5:**
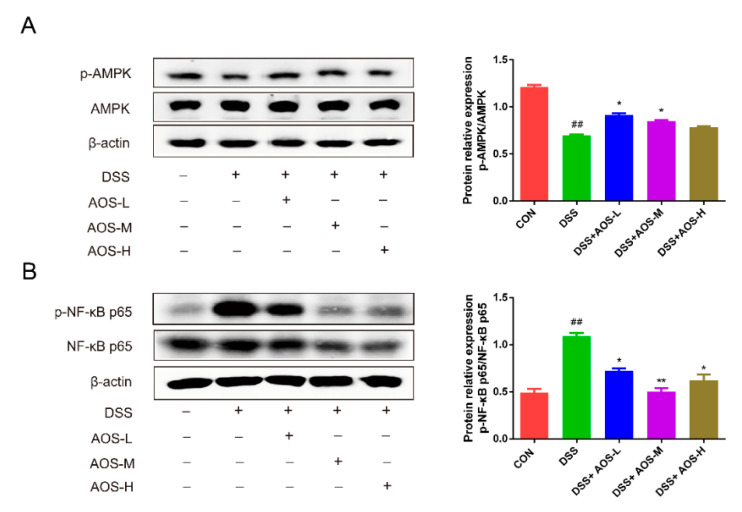
Effects of AOS on DSS-induced inflammation in colonic tissues. (**A**) Western blotting analysis of AMPK and p-AMPK levels in colonic tissues, with p-AMPK/AMPK being analyzed with ImageJ software. (**B**) Western blotting analysis of NF-κB p65 and p-NF-κB p65 levels in colonic tissues, with p-p65/p65 being analyzed with ImageJ software. ^##^ *p* < 0.01 vs. Control group; * *p* < 0.05, ** *p* < 0.05 vs. LPS group; n = 3.

Previous studies have shown the involvement of AMPK in suppressing NF-κB activation and inflammation [37]. The potential mechanisms of AOS in alleviating inflammation were further explored by analyzing AMPK and NF-κB phosphorylation in AOS-pretreated mice with colitis. In Figure 5A, DSS challenge was seen to decrease AMPK phosphorylation in colonic tissues (^##^ *p* < 0.01 vs. Control group), but this decrease was curbed by AOS administration. Compared with the DSS group, the AOS-M group showed a significant increase in p-AMPK levels (*p* < 0.05). In Figure 5B, DSS-induced NF-κB activation was observed in colonic tissues, as evidenced by the increased p-p65 levels (^##^ *p* < 0.01 vs. Control group). However, the p-p65 levels showed a remarkable decrease in all the three AOS groups versus the DSS group (* *p* < 0.05, ** *p* < 0.01 vs. DSS group).

### 3.6. AOS Suppressed LPS-Induced Inflammation In Vitro through AMPK-NF-κB Signaling Pathway

**Figure 6:**
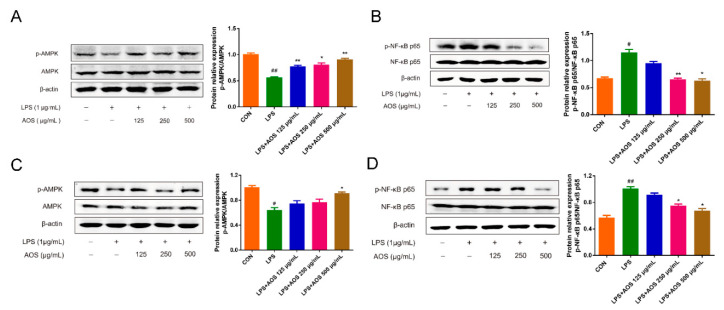
Effects of AOS on LPS-induced inflammation in RAW 264.7 and BMDM cells. RAW 264.7 and BMDM cells were pretreated separately with AOS at 125, 250, and 500 μg/mL for 24 h, followed by 1 h stimulation with 1 μg/mL LPS. Western blotting analysis of AMPK and p-AMPK levels in RAW 264.7 cells (**A**) and BMDMs (**C**), with p-AMPK/AMPK being analyzed with ImageJ software. Western blotting analysis of NF-κB p65 and p-NF-κB p65 levels in RAW 264.7 cells (**B**) and BMDMs (**D**), with p-p65/p65 being analyzed by ImageJ software. * *p* < 0.05, ** *p* < 0.01 vs. LPS group; ^#^ *p* < 0.05, ^##^ *p* < 0.01 vs. Control group; n = 3.

Next, we investigated the effects of AOS on AMPK and NF-κB phosphorylation in LPS-stimulated RAW 264.7 and BMDM cells in vitro. In Figure 6A, AMPK phosphorylation level was seen to be significantly downregulated under 1 h LPS stimulation (^##^ *p* < 0.01 vs. Control group), but AOS pretreatment (125, 250, and 500 μg/mL) could significantly upregulate p-AMPK levels in different degrees (* *p* < 0.05, ** *p* < 0.01 vs. LPS group). After 1 h LPS administration, NF-κB phosphorylation level showed a marked increase in RAW 264.7 cells (^##^ *p* < 0.01 vs. Control group), but this increase was significantly blocked by AOS pretreatment (Figure 6B). Specifically, 250 and 500 μg/mL AOS pretreatment could significantly suppress the p-p65 level (* *p* < 0.05, ** *p* < 0.01 vs. LPS group), and 125 μg/mL AOS pretreatment could also inhibit p65 phosphorylation, but not significantly. In Figure 6C,D, AOS treatment was also seen to increase p-AMPK level and inhibit NF-κB phosphorylation in LPS-stimulated BMDMs (* *p* < 0.05, ** *p* < 0.01 vs. LPS group). These results indicated that AOS could inhibit LPS-induced inflammation by inhibiting NF-κB signaling and activating AMPK signaling.

### 3.7. AOS Supplementation Beneficially Modulated Gut Microbiota

**Figure 7:**
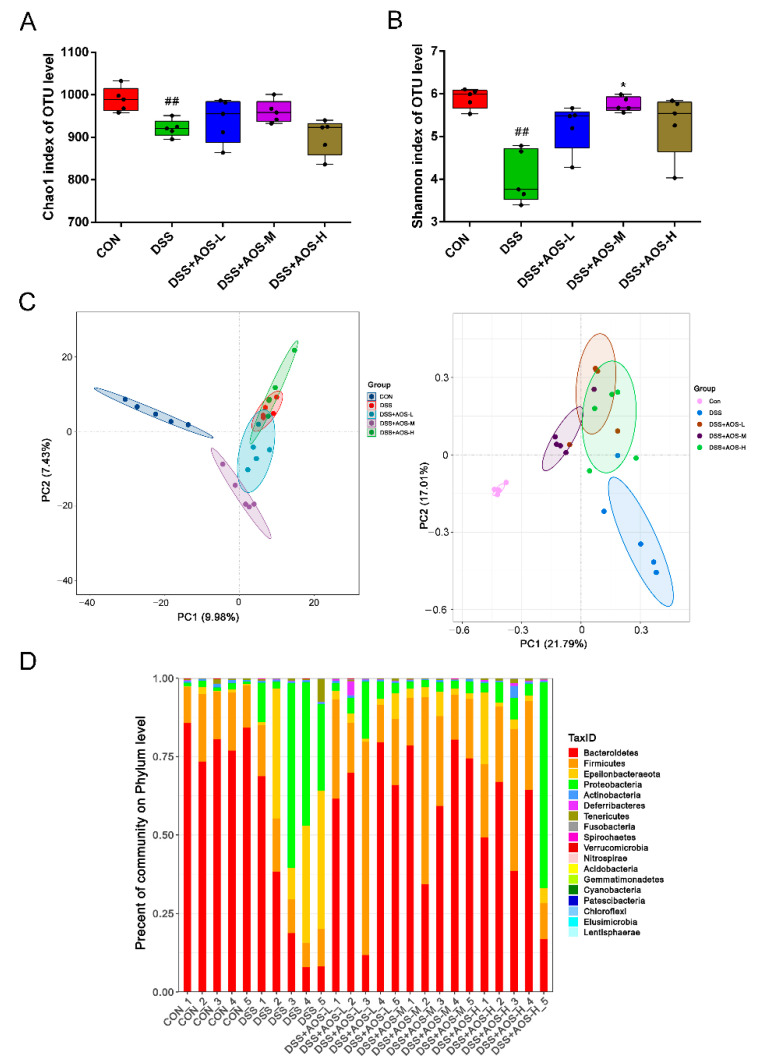
Effects of AOS on gut microbiota overall structure as detected by gut microbiota composition analysis through high-throughput sequencing of 16S rRNA genes. (**A**) Alpha diversity measured by Chao1. (**B**) Alpha diversity measured by Shannon index. (**C**) Beta diversity evaluated by PCA and PCoA analyses. (**D**) Bacterial taxonomic profiling at phylum level. * *p* < 0.05 vs. DSS group; ^##^ *p* < 0.01 vs. Control group; n = 5.

The effects of AOS on gut microbiota were explored by high-throughput sequencing analysis of bacterial 16S rRNA genes in colonic contents. Here, α-diversity indices, including the Chao index and Shannon index, were used to assess the abundance and diversity of microbiota. In Figure 7A,B, DSS treatment was seen to significantly reduce gut microbiota abundance and diversity (^##^ *p* < 0.01 vs. Control group), while AOS administration could improve gut microbiota abundance and diversity to a varying degree. Among the three AOS groups, only the AOS-M group was significantly higher than the DSS group in the Shannon index (*p* < 0.05). Based on principal coordinates analysis (PCoA) and Principal component analysis (PCA), the five groups showed a clearly discernible clustering separation. In Figure 7C, DSS treatment was seen to cause marked shifts in gut microbial community structure. As shown by β-diversity analysis (Figure 7C), the AOS-M group was closer to the Control group than the DSS group in species distribution. Furthermore, exposure to AOS altered the relative abundance of bacterial community composition at phylum level (Figure 7D). Compared with the Control group, DSS treatment could increase the relative abundance of Proteobacteria and Epsilonbacteria and decrease the relative abundance of Bacteroidetes. These changes could be reversed by AOS intervention.

**Figure 8:**
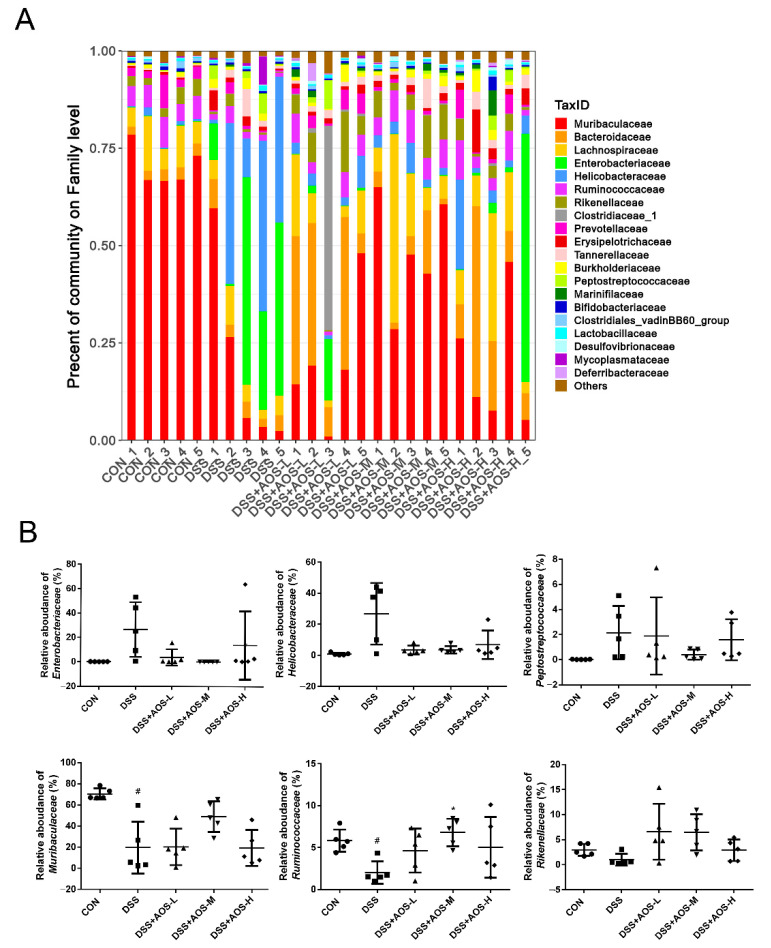
Effects of AOS on the gut microbial composition of colonic contents at family level. (**A**) Bacterial taxonomic profiling at family level. (**B**) Relative abundance of gut microbiota at family level. ^#^ *p* < 0.05 vs. Control group, * *p* < 0.05 vs. DSS group, n = 5.

Exposure to AOS could alter the relative abundance of bacterial community composition at family level (Figure 8A). In Figure 8B, it was shown that, compared with the Control group, the DSS group showed an increase in the proportions of Enterobacteriaceae, Helicobacteraceae, and Peptostreptococcaceae and a significant (*p* < 0.05) decrease in the proportions of Muribaculaceae and Ruminococcaceae, coupled with a slight but not significant decrease in Rikenellaceae relative abundance. However, AOS administration could partly restore the proportions of these bacteria, resulting in a higher (*p* < 0.05) relative abundance of Ruminococcaceae in the AOS-M group than in the DSS group and a slightly lower relative abundance of Enterobacteriaceae, Helicobacteraceae, and Peptostreptococcaceae in the AOS-M group than in the DSS group (Figure 8B).

**Figure 9:**
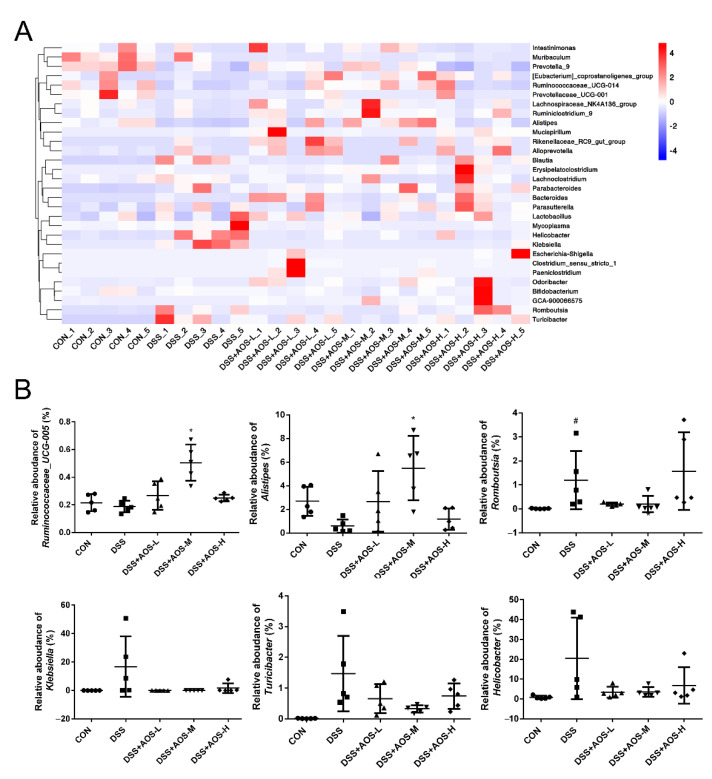
Effects AOS on the gut microbial composition of colonic contents at genus level. (**A**) Heat map analysis of gut microbial composition at genus level. (**B**) Relative abundance analysis of gut microbiota at genus level. * *p* < 0.05 vs. DSS group; ^#^ *p* < 0.05 vs. Control group; n = 5.

At the genus level, the community composition was displayed intuitively through the heatmap visualization method (Figure 9A). In Figure 9B, compared with the Control group, the DSS group was lower in the proportion of Alistipes, higher in the proportion of Romboutsia (*p* < 0.05), and slightly but not significantly higher in the proportion of Klebsiella, Turicibacter, and Helicobacter. Compared with the DSS group, the AOS-M group showed an increase (*p* < 0.05) in the proportions of Ruminococcaceae_UCG-005 and Alistipes and a slight but not significant decrease in the proportions of Romboutsia, Klebsiella, Turicibacter, and Helicobacter.

## 4. Discussion

This study focused on the effects and mechanism of AOS on colitis amelioration. AOS were shown to exert anti-inflammatory effects in DSS-challenged mice and LPS-stimulated macrophages by activating the AMPK signaling pathway and modifying gut microbiota composition.

Our results showed that 125–500 μg/mL AOS (DP: 2–4) did not cause cytotoxicity in LPS-treated RAW 264.7 cells, but AOS could significantly inhibit LPS-stimulated NO production, implying that the AOS inhibitory effect on NO production was not attributed to cytotoxicity. Moreover, AOS could significantly downregulate IL-1β, IL-6, and TNF-α levels and increase IL-10 release in LPS-exposed RAW 264.7 and BMDM cells. This was consistent with the study of Zhou et al., who reported that guluronate oligosaccharides derived from oxidative degradation (GOS-OD) could reduce the secretion of TNF-α, IL-1β, and IL-6 in LPS-activated RAW 264.7 cells by blocking the activation of NF-κB and mitogen-activated protein kinase (MAPK) pathways [6].

Several recent studies have confirmed the protective effect of AOS on intestinal injury and inflammation caused by toxins. Li et al. [17] showed the protective potential of AOS against FB1-induced ileal and colonic damage in mice by improving mucosal layer integrity, reducing cell apoptosis, attenuating gut dysbiosis, and alleviating inflammation and oxidative stress. Additionally, AOS could decrease the concentrations of IL-1β, IL-6, TNF-α, and IFN-γ and increase IL-10 levels in the plasma of FB1-treated mice. Moreover, AOS showed antioxidant capacity in FB1-treated mice by enhancing plasma T-SOD levels and decreasing plasma MDA levels. In the study of Wan et al. [18], AOS were shown to ameliorate ETEC-induced jejunal injury in weaned pigs by decreasing jejunal TNF-α and IFN-γ concentrations and increasing jejunal occludin protein abundance. In the study of He et al. [38], UAOS were found to attenuate DSS-induced colitis in mice by reducing the expression of inflammatory factors (COX-2 and TNF-α) and upregulating the expression of tight-junction proteins (ZO-1 and occludin). In the present study, AOS intervention was shown to significantly prevent colon shortening, body weight loss, rectal bleeding, and DAI scores, resulting in reduced ileal and colonic damages, low levels of IL-1β, IL-6, TNF-α and IFN-γ, and high levels of IL-10 in AOS-treated mice, suggesting that AOS could inhibit DSS-induced ulcerative colitis.

Inflammation was known to reduce AMPK activity through multiple mechanisms [20]. Consistently, AMPK phosphorylation was shown to be significantly decreased in LPS-stimulated RAW 264.7 cells [39,40,41] and in the colonic tissues of DSS-induced colitis mice [42]. However, AMPK activation, which is initiated by phosphorylation, exerts anti-inflammatory activities in immune cells such as macrophages, neutrophils, T cells, and mast cells [26,43,44]. AMPK activator administration has been confirmed to attenuate DSS-induced colitis in mice [42,45]. Like previous studies [39,40], our results showed that AMPK phosphorylation was downregulated in macrophages in response to 1mh LPS treatment and p-AMPK level was decreased in the colonic tissues of DSS-induced mice, but AOS could induce AMPK activity in vivo and in vitro by increasing AMPK phosphorylation.

Accumulating evidence indicated that AMPK signaling activation could downregulate NF-κB function through several downstream targets, including kappa B kinase inhibitor (IKK) [46], silent information regulator 1 (SIRT1) [47], Forkhead Box O (FoxO) family [48], p53 [49], and peroxisome proliferator-activated receptor-gamma coactivator-1alpha (PGC-1α) signaling [37,50]. Activating the NF-κB signaling pathway in macrophages could induce the expression of various proinflammatory cytokines, including TNF-α, IL-1β, IL-6, etc. As previously reported, AOS could produce anti-inflammatory effects in LPS-stimulated IPEC-J2 cells and ETEC-infected weaned pigs by suppressing NF-κB activation [18]. Here, our results indicated that AOS administration could significantly increase AMPK phosphorylation and inhibit NF-κB p65 activation in colonic tissues, resulting in a reduction of proinflammatory factor levels and gut injury severity in DSS-induced colitis mice.

Intestinal microflora balance is crucial to maintain the physiological function of the host, including the absorption of nutrition, the development of mucosal immunity, the formation of an intestinal epithelial barrier, and the resistance of pathogens [51]. IBD patients were found to have a reduced microbial diversity in inflamed colons [52]. Consistent with a previous report [53], our study showed a decrease in alpha diversity in DSS-treated mice, as indicated by Shannon and Chao1 indexes. Furthermore, AOS administration could increase intestinal microbiota diversity, enabling clear clustering of microbiota in each group in PCoA analysis.

Previous studies found a significant decrease in the proportion of *Bacteroidetes* in IBD patients and DSS-induced colitis mice [54,55,56]. In the present study, DSS treatment was also found to cause a decrease in *Bacteroidetes* abundance at the phylum level. Moreover, DSS treatment resulted in an increase in the relative abundance of *Proteobacteria* and *Epsilonbacteraeota,* which was consistent with two previous studies [57,58]. The expansion of *Proteobacteria*, a microbial signature of microbiota disorder, is associated with epithelial dysfunction [59,60]. *Proteobacteria* can cause inflammation and alter gut microbiota, thereby promoting IBD development [61]. Our study showed that AOS intervention could reverse and shift microbiota disorder to levels close to the control group. Our results agreed with a recent study on UAOS antiobesity effect in that UAOS treatment could increase *Bacteroidetes* relative abundance and decrease *Proteobacteria* relative abundance.

At the family level, the DSS-group microbiota was characterized by high abundances of pathogenic bacteria *Enterobacteriaceae*, *Helicobacteraceae*, and *Peptostreptococcaceae* and low abundances of *Muribaculaceae*, *Ruminococcaceae*, and *Rikenellaceae*. *Enterobacteriaceae* is the typical family of *Proteobacteria* and is traditionally associated with IBD development. The abundance of *Enterobacteriaceae*, *Peptostreptococcaceae*, and *Helicobacteraceae* was reported to increase significantly in IBD patients [62,63]. DSS was also shown to induce an abundance increase of *Enterobacteriaceae* [32], *Helicobacteraceae* [64], and *Peptostreptococcaceae* [65]. *Muribaculaceae*, the dominant family in the intestine of mice, is involved in the degradation of complex carbohydrates [66]. *Muribaculaceae* was reported to be positively correlated with propionate production [67], which is known to improve intestinal epithelial health. *Ruminococcaceae* is widely recognized as probiotics owing to the capability of producing short-chain fatty acids (SCFAs), especially butyric acid. Compared with healthy volunteers, IBD patients have a lower abundance of *Ruminococcaceae* [68]. *Ruminococcaceae* and *Rikenellaceae* were reported to be negatively associated with proinflammatory cytokine levels in colitis mice [69]. The abundance of *Muribaculaceae* [56], *Ruminococcaceae* [32], and *Rikenellaceae* [70] was reported to decrease remarkably in DSS-treated colitis mice. Our data confirmed that the gut microbiota dysbiosis in DSS-induced colitis mice could be recovered by AOS supplementation.

At the genus level, AOS-M treatment could significantly enrich several SCFAs-producing bacteria, including *Ruminococcaceae_UCG-005* and *Alistipes*. *Ruminococcaceae_UCG-005*, belonging to the family *Ruminococcaceae*, is mainly involved in fiber degradation and butyrate production. SCFAs-producing bacteria can provide energy sources for enterocytes, protect intestinal mucosa integrity, regulate immunity, and reduce inflammation. The role of *Alistipes* in gut inflammation is controversial. Most studies supported that *Alistipes* has a protective effect in colitis [71,72,73] and that *Alistipes finegoldii* addition could ameliorate DSS-induced colitis in mice [74]. Meanwhile, several studies confirmed a highly negative association of *Alistipes* abundance with inflammatory cytokine levels [72,73].

Furthermore, in the present study, the relative abundances of *Romboutsia*, *Klebsiella*, *Turicibacter*, and *Helicobacter* were increased by DSS treatment, but most of these changes were reversed by AOS administration. The role of *Romboutsia* in gut inflammation remains unclear. In some studies, *Romboutsia* was considered as beneficial bacteria in acute colitis [75], but its enrichment was in DSS-induced chronic colitis [76,77] and azoxymethane (AOM)/DSS-induced colitis-associated cancer (CAC) [78]. Consistent with an aforementioned study [78], our results showed that DSS could increase *Romboutsia* abundance. *Klebsiella*, a pathogen belonging to the family *Enterobacteriaceae*, was significantly more abundant in the gut of mice with DSS-induced colitis [79,80] and played a key role in pathological damage initiation and perpetuation in various intestinal inflammations and cancers [57,81,82]. *Turicibacter* was reported to be enriched in the intestine of DSS-treated mice [64,83,84] and have a positive correlation with proinflammatory cytokine levels [53,85]. *Helicobacter* spp. was proven to be a typical pathogen in IBD patients [86], and *Helicobacter* infection could induce IBD in immunodeficient mice [87].

## 5. Conclusions

This study demonstrated that AOS could inhibit LPS-mediated inflammatory response and attenuate DSS-induced colitis through AMPK signaling activation and NF-κB activation suppression. AOS supplementation could recover dysbiosis in mice with colitis by enriching the potential probiotic bacteria such as *Muribaculaceae*, *Ruminococcaceae*, and *Rikenellaceae* and inhibiting the proliferation of detrimental bacteria such as *Enterobacteriaceae*, *Helicobacteraceae*, and *Peptostreptococcaceae*. Our results suggest the potential use of AOS as a therapeutic agent or nutraceutical against intestinal inflammatory injury.

## Data Availability

The data obtained in this study are available on request from the author.
